# Analysis of PBT and PET cyclic oligomers in extracts of coffee capsules and food simulants by a HPLC-UV/FLD method

**DOI:** 10.1016/j.foodchem.2020.128739

**Published:** 2021-05-30

**Authors:** Joao Alberto Lopes, Emmanouil D. Tsochatzis, Lubomir Karasek, Eddo J. Hoekstra, Hendrik Emons

**Affiliations:** a European Commission, Joint Research Centre (JRC), Geel, Belgium; b European Commission, Joint Research Centre (JRC), Ispra, Italy

**Keywords:** Polyesters cyclic oligomers, Coffee capsules, Non-intentionally added substances (NIAS), Migration testing, Exposure assessment

## Abstract

A HPLC-UV/FLD method was validated for the quantification of six polyethylene terephthalate (PET) and four polybutylene terephthalate (PBT) oligomers. PBT oligomers are EU regulated, while the PET ones are considered non-intentionally added substances (NIAS). LOQs were higher than 0.4 and 3.5 μg kg^−1^ for the simulants and in the polymer extracts, respectively. Recoveries ranged from 95 to 114 % with RSDs below 12%. Migration testing of PBT and polypropylene coffee capsules were performed with H_2_O and simulant C, and extracts were obtained with accelerated solvent extraction (ASE). For the latter legislative limits weren’t surpassed. As no migration limits are existing for the analytes, both EFSA’s toxicological threshold of concern (TTC) and sum of oligomers approaches were applied. The majority of oligomers were below the TTC (90 µg/person/day), but the limit value of 50 µg/kg food was surpassed for some capsules, which indicates a significant intake in both single and multiple consumption.

## Highlights

• PET and PBT cyclic oligomers in food contact materials (plastic coffee capsules).
• Validation and application of a HPLC-UV/FLD for quantification of the studied PET and PBT oligomers.
• Method validation for 1st Series PET cyclic oligomers and PBT, with proper analytical standards, with known purity.
• Exposure assessment based on TTC and EFSA scientific opinions.

## 1 Introduction

Polyesters (PES) are a group of plastics that are used nowadays in many applications and account for almost 18% of the world polymer production (Ting Wu, Hu, Du, Jiang, & Yu, 2014). Among the most relevant PES are polyethylene terephthalate (PET), polybutylene terephthalate (PBT), and polytrimethylene terephthalate (PTT). PET is selected for food contact materials (FCMs) due to its impact resistance, strength, flexibility and resistance to high temperatures (Ting Wu et al., 2014; Hoppe, Fornari, de Voogt, & Franz, 2017). Since recently PBT is also used in FCM applications such as coffee capsules, oxygen-barrier films, kitchenware, microwaveable dishware (Brenz et al., 2017, Brenz et al., 2018), whereas the use of PTT still remains restricted to the textile and automotive industry (Piccinini, Senaldi, & Alberto Lopes, 2014).

Coffee brewed from coffee capsules systems is slowly replacing traditional brewing methods. Coffee capsules are small recipients containing previously roasted and ground coffee beans that are used in specially designed systems. These capsules are normally fabricated in polypropylene (PP) and aluminium, but some other materials such as PET and PBT can also be found in the market. Various configurations and brands are available commercially, but coffee capsules normally include inner layers for the prevention of oxidation and humidity loss, filters for the elimination of coffee particles and covers sealed to the capsule base. All these parts can be produced from many different materials. After placing the capsule in the coffee system, the capsule is pierced (from the top or the bottom) and hot pressurised water is passed through in order to obtain a coffee beverage. The market for the coffee capsules and respective systems is increasing, with some estimations foreseeing a total market value of around 30 billion euros by 2025 (Global Coffee Pods and Capsules Market - Growth, Trends and Forecasts (2020–2025)).

During polymerization oligomers are formed as side-reaction products. These oligomers can range from simple dimers up to decamers in both cyclic and linear structures. Their formation seems to be unavoidable and even thermodynamically favoured (Brenz et al., 2017, Goodman and Nesbitt, 1960).

PET oligomers are considered as non-intentionally added substances (NIAS) and have shown to migrate from the plastic material into the food (Brenz et al., 2017, Eckardt et al., 2019, Genualdi et al., 2014, Heimrich et al., 2015; Kappenstein et al., 2018, Tsochatzis et al., 2020). A mixture of some PBT cyclic oligomers (dimer to pentamer) is included in the positive list of Regulation (EU) No. 10/2011 as FCM substance No. 885 (EC, 2011, EFSA, 2009), but its use as additive is restricted to FCMs made of PET, PBT, polycarbonate, polystyrene and rigid poly(vinyl chloride) in concentrations up to 1% w/w. It shall only come in contact with aqueous, acidic and alcoholic foods for long term storage at room temperature. Currently there are no migration limits for PET and PBT oligomers.

Toxicological data for most known oligomers are not existing, making it very difficult to perform a risk assessment. The European Food Safety Authority (EFSA) published several opinions indicating a limit of 0.05 mg kg^−1^ food for the sum of the total oligomeric fraction below 1000 Da migrating from new co-polymers to food (EFSA, 2014b, EFSA, 2012, EFSA, 2014a, EFSA, 2019). EFSA also proposed the application of the Threshold of Toxicological Concern (TTC) approach for classifying substances for which no toxicity data are available and no genotoxicity is expected (EFSA, 2012, EFSA, 2016). *In silico* toxicological assessment of PET oligomers was previously reported, without highlighting any genotoxicity alerts (Tsochatzis et al., 2020). According to the TTC concept, all the studied PBT and PET cyclic oligomers are considered as belonging to Cramer Class III, implying a potential significant toxicity and a maximum consumption limit of 90 μg/person/day (Cramer et al., 1978, EFSA, 2014b, EFSA, 2012, EFSA, 2014a, EFSA, 2019).

A limited number of studies on the analysis of PET and PBT oligomers in FCMs have been reported so far and are summarised in Table 1.

**Table 1:** Relevant studies on the analysis of PET and PBT oligomers in FCMs.

Oligomers	FCMs	Matrix	Analytical technique	Ref.
PET cyclic oligomers	Microwavable PET	Migration solutions	HPLC-FLD	Begley et al., 1990
PET cyclic and linear oligomers	Ovenable PET bags and trays	Migration solutions	HPLC-UV	De Freire, Damant, Castle, & Reyes, 1999
PES linear and cyclic oligomers	Several PES items	Extraction solutions after hydrolysis	HPLC-UV and GC–MS LC-ESI-MS	Brenz et al., 2017
PES cyclic oligomers	FCM multilayers	Migration solutions	UPLC-MS-qTOF	Ubeda, 2017
PET linear and cyclic oligomers	Virgin and recycled PET food-grade pellets	Extraction and migration solutions	UPLC-MS-qTOF	Ubeda, Aznar, & Nerin, 2018
PBT linear and cyclic oligomers	Several PBT items	Extraction solutions	HPLC-UV and LC-ESI-MS	Brenz et al., 2018
PET and PBT cyclic dimers and trimers	PET bottles	Migration solutions	HPLC-UV	Tsochatzis et al., 2019a; Dehouck et al., 2018
PBT cyclic dimer to pentamer	PBT beverage cups	Migration and extraction solutions	HPLC-UV	Tsochatzis et al., 2019b
PET trimer and PBT dimer and trimer	PET and PBT food-grade pellets	Extraction solutions	HPLC-UV	Eckardt et al., 2019
PES cyclic oligomers	PLA pellets and films	Migration and total dissolution solutions	UPLC-qTOF-MS	Ubeda, 2019
PET cyclic and linear oligomers	PET tea bags	Migration solutions	UHPLC-qTOF-MS	Tsochatzis et al., 2020
PES cyclic oligomers	FCM multilayers	Migration solutions	UPLC-qTOF-MS	Ubeda, 2020

As only very few commercially analytical standards of PET and PBT oligomers were available until recently, most studies had to rely on the semi-quantification of oligomers. That is the case of all the works included in Table 1, with the exception of Tsochatzis et al., 2020. Frequently the PET cyclic trimer was used as a universal calibrant for the quantification of all the oligomers of whatever chemical structure. However, dimer to heptamer PET cyclic oligomers were used in a recent study on their migration from tea bags (Tsochatzis et al., 2020).

In this work an analytical method for the quantification of cyclic PET (dimer to heptamer) and PBT (dimer to pentamer) oligomers by HPLC-UV/FLD has been developed. The method was validated using analytical standards of known purity for all the ten oligomers and has been applied to their quantification in extracts of PBT and PP coffee capsules using three different extraction techniques. A quantification of oligomers migrating into food simulants was also performed. This allowed making a first estimation of the potential exposure to PET and PBT oligomers based on five different types of commonly used coffee capsules. The risk was assessed applying two approaches used by EFSA, namely the TTC and a sum limit used for similar oligomers of other polyesters.

## 2 Materials and methods

### 2.1 Chemicals

Ethanol and 1,1,1,3,3,3-hexafluoro-2-propanol (HFIP) (Chromasolv grade), trifluoroethanol (TFE) (for HPLC, ≥ 99.0%), acetonitrile and formic acid (LC-MS grade) were supplied by Sigma Aldrich (Steinheim, Germany). Ultrapure water (MilliQ, 18.2 MΩ) was used in the preparation of solutions. All the PET cyclic oligomers were supplied by TRC Chemicals (Toronto, Canada) with stated purities ranging from 95% (tetramer) to 98% (dimer) w/w. Dimer to pentamer PBT cyclic oligomers were isolated using a preparative HPLC methodology on a raw polymeric mixture from JRC's reference collection of FCM substances, a sample provided by the petitioner of FCM No. 885 of Commission Regulation (EU) No. 10/2011 (Regulation (EU) No. 10/2011). The PBT oligomers were in-house characterised and checked for purity, which was found to be 96% w/w or higher (Tsochatzis et al., 2019c). All chemical information for the PET and PBT cyclic oligomers is given in Table S1 of the supplementary information.

### 2.2 Preparation of standard solutions

Stock solutions containing 100 μg mL^−1^ of each oligomer were prepared gravimetrically from analytical standards of the oligomers considering their purity and using HFIP as solvent. Stock solutions were stored at −20 °C and were stable for at least 4 months at 4 °C. Appropriate working solutions were then prepared by dilution with acetonitrile and stored at 4 °C. Fresh working standards were prepared every week. Both stock and working standard solutions were prepared in amber vials in order to prevent light-induced degradation or isomeric conversion.

### 2.3 Samples

Single-use coffee capsules from 5 different brands were bought at local supermarkets. All the capsules were carefully opened, disassembled, emptied of coffee and cleaned gently with a cloth. It appeared that the adhesives remained intact so it was possible to close the empty capsules with the upper external aluminium lid as if they were unused. The capsules were then stored under dry conditions wrapped in aluminium foil (room temperature, 20.0 ± 2.5 °C).

A verification of the type of materials used in the capsules was performed by Attenuated Total Reflection-Fourier Transform Infra-Red (ATR-FTIR) spectroscopy and Differential Scanning Calorimetry (DSC). Recorded spectra and thermograms were compared with the in-house database. Two capsules consisted of PBT (PBT caps. 1 and 2) and three of PP (PP caps. 1, 2 and 3). They all contained an external aluminium lid, a PET/aluminium inner film at the bottom just above a PET grid filter, and another PET film at the top. The PET films are used to maximise the preservation of coffee during storage time, and the PET/aluminium bottom filter grid to prevent coffee particles ending in the solution. Only the PP caps 2 had a different configuration, with only one PET film, but it contained a soluble herb-coffee beverage.

### 2.4 Instrumentation

#### 2.4.1 Differential Scanning Calorimetry (DSC)

DSC analyses were performed with a TA Instruments Model Q100 (Newcastle, DE, USA) equipped with an autosampler. The temperature control program consisted of heating from 0 °C to 300 °C at 20 °C/min (1st cycle), cooling to 0 °C at 20 °C/min (2nd cycle) and heating back to 300 °C at 20 °C/min. The heating scans were performed with the sample under a constant flow of nitrogen at 50 mL min^−1^.

#### 2.4.2 Fourier-transform infrared spectroscopy (FTIR)

All spectra were acquired with a diamond crystal Attenuated Total Reflectance (ATR) FTIR spectrometer (Perkin Elmer Spectrum 2000). Spectra were acquired in the scan range 4000.00–530.00 cm^−1^, with a resolution of 4.00 cm^−1^ and a total of 8 scans for each sample that were averaged to eliminate background noise. Samples were analysed without any preparation and the obtained spectra were compared with a spectral database.

#### 2.4.3 Accelerated solvent extraction (ASE)

An ASE extraction system Dionex ASE350 (Dionex Corporation, Sunnyvale, CA, USA) was used for the extraction with the capsules. Stainless-steel ASE cells of 10 mL, cellulose inner filters and inert glass spheres as cell fillers were used. The extraction was performed with ethanol at an oven temperature of 100 °C and a 5 min heat-up time under a pressure of 1500 psi. Five static cycles were used with a static time of 3 min/cycle. The cell was finally rinsed with ethanol (60% of cell volume) and purged with nitrogen for 60 s (125–150 psi) to obtain a final volume of 30 mL of extract.

#### 2.4.4 Chromatographic system

The chromatographic system consisted of an Agilent Technologies 1200 series HPLC system 137 (Waldbronn, Germany), equipped with a thermostatic column compartment, an autosampler, an UV detector and an FLD. The separation was carried out on the chromatographic column Agilent Zorbax Eclipse XDB-C18 (150 × 4.6 mm, 5 μm), which was thermostated at 40 °C. The gradient elution program consisted of a mixture of acetonitrile (A) and ultrapure water (B) at a flow of 2 mL/min. The chromatographic run started with an isocratic step of 50% B until 4 min, followed by a gradient decrease of B to 25% until 13 min, followed by an isocratic step until 17 min and a gradient decrease to 5% till 22 min. After an isocratic step with 5% of B from 22 to 24 min, the gradient decreased to the initial conditions followed by an equilibration time of 4 min. The injection volume was set to 50 μL. Maximum UV wavelengths for the target analytes were studied and 240 nm was selected as the best compromise (data not shown). For the FLD detection, a number of tests have been performed (see section 3.1). Due to the different responses for the 10 oligomers, two FLD channels were selected to be used simultaneously with an excitation at 250 nm and emission at both 320 nm and 360 nm.

### 2.5 Extraction of the analytes from coffee capsules

Different solvents were tested for the extraction of the single-use coffee capsules in order to establish the initial content of the existing oligomers and to assess compliance with Regulation (EU) No. 10/2011 for additive FCM No. 885. ASE was compared with Soxhlet and solid–liquid extraction (SLE) in order to establish the most efficient, comparable and applicable method. One type of the PBT coffee capsules was selected as test item in order to perform the comparison of methods. The coffee and all inner parts including the sealing films made of PET were removed. Ethanol was selected as the extraction solvent.

Before extraction, the coffee capsule was cut into small pieces, which were ground in a centrifugal mill (Mill ZM 200, Retsch) with dry ice to obtain a powder with a particle size of 500 µm. This sample was then homogenised and divided into three subsamples of about 1 g. Each subsample was used with one type of extraction method. For the Soxhlet extraction, the subsample was inserted in a cellulose extraction cartridge. The volume of ethanol used was 100 mL and the extraction lasted for 48 h. An additional 24 h extraction was performed to confirm that no oligomers could still be extracted.

For ASE, the subsample was mixed thoroughly with 2 g of inert glass spheres and placed into a 10 mL stainless steel cell containing a cellulose filter.

Regarding SLE, the subsample was inserted in a 30 mL amber vial and filled with ethanol. The vial was sonicated at 40 °C for 2 h and then left under agitation during 48 h.

All extracts (Soxhlet, ASE and SLE) were filled-to-volume (100 mL) and filtered through PTFE 0.22 μm filters. Injection samples were prepared by concentrating (rotatory evaporator, 40 °C) the initial volume 1:10 followed by re-filtering.

### 2.6 Migration and experiments mimicking potential daily uptake

When a coffee capsule is placed in the appropriate instant coffee machine, water is coming into contact with the polymer and the ground coffee at an average temperature of 80 °C to 85 °C (according to manufacturer manuals) during some seconds. As there are no official tests described for this type of FCM, two experiments have been designed for their analysis. In the first one the capsules were studied mimicking the preparation of a normal coffee with an instant coffee machine. This type of devices are generically consisting of an external tank, from which water is pumped through an internal electric heater to a dispenser placed at the top of the machine. The heated water then flows through a capsule placed and pierced in a sample holder in order to obtain the coffee beverage, collected in an external recipient. In this work, to have the solutions as simple as possible (free of interfering substances from the coffee itself), an empty pre-cleaned coffee capsule was inserted into the system (inner parts were kept inside the capsule). Then either MilliQ water (an approximation to drinking water) or food simulant C (containing 20% ethanol, official food simulant for coffee, according to Regulation (EU) No. 10/2011) were passed through the system. Around 100 mL, i.e. a volume between an expresso and a cappuccino (according to the manufacturer description), were collected, adjusted to a volume of 100 mL and filtered through PTFE 0.22 μm filters. The collected migration samples were concentrated in a rotary evaporator to a volume of about 5 mL, transferred into a volumetric flask and adjusted to 5 mL. An aliquot of 1 mL was then filtered (0.22 μm PTFE filter) and placed under a gentle stream of nitrogen at 45–50 °C. After evaporation to a volume of 0.75 mL a mixture of HFIP/TFE/ethanol (2:2:4, v/v/v) was added up to a final volume of 1 mL.

In the second test, an adapted migration experiment was performed (Alberto Lopes, Tsochatzis, Robouch, & Hoekstra, 2019). The coffee capsules were used for an immersion test with both water and food simulant C. Each pre-emptied capsule has been immersed, with all the inner parts, e.g. sealing films, in 100 mL of each liquid and left at boiling temperature (92–93 °C in water and 84–85 °C in food simulant C) for 5 min, the minimum test contact time in the Regulation (Regulation (EU) No. 10/2011). The post-sample-treatment was the same as in the coffee machine test.

### 2.7 Method performance characterisation

To evaluate the method’s fitness-for-purpose the following performance characteristics were studied: linearity, precision, trueness, limit of detection (LOD) and quantification (LOQ), following guidelines for performance criteria/validation of analytical methods (Thompson et al., 2002, Bratinova et al., 2009, DG Sante, 2017). The linearity of the signal-mass fraction plot was evaluated by analysing water and food simulant C fortified with standard solution mixtures of the 10 oligomers at 7 levels (from 0.02 to 2000 μg kg^−1^). For the transformation of concentrations (μg L^-1^) to mass fractions (μg kg^−1^) in water and food simulant C (20% v/v ethanol in water), a density of 1.0 g mL^−1^ was used. Linear regression coefficients (R^2^) and residuals plot were examined to assess linearity. Estimations of LOD and LOQ were based on the signal (S) to noise (N) ratio (S/N). Mean value and standard deviation of the S/N were obtained from 5 chromatograms of the lowest calibration level for each oligomer (H_2_O and food simulant C). The noise has been checked at different points of the chromatogram’s baselines to ensure a constant behaviour. The LOD was estimated as analyte concentration providing an S/N of 3, while the LOQ was calculated as 3 times the LOD. (Thompson et al., 2002, Bratinova et al., 2009, DG Sante, 2017)

For the trueness evaluation a recovery study was performed, while the precision has been evaluated in terms of repeatability and intermediate precision by evaluating the relative standard deviations (RSD). For the repeatability assessment, 6 replicates were analysed during one day, while for the intermediate precision 6 replicates were analysed on three consecutive days. The trueness estimate was based on the calculation of the recovery as relative amount found in the fortified water or food simulant C divided by the known amount added, at 2 different concentrations.

### 2.8 In silico toxicological classification of the analytes

The absence of genotoxicity alerts for PET cyclic oligomers was recently reported (Tsochatzis et al., 2020). No data are available for the PBT cyclic oligomers. Based on the structural similarities between both types of oligomers a similar toxicological behaviour was assumed here. Therefore, the TTC concept has been applied. The estimation of the theoretical toxicity of the cyclic PET and PBT oligomers was performed using the software Toxtree v2.6.13, based on their chemical structure and following the respective Cramer rules with extensions (EC, 2018, Ltd, 2011). Thereby, toxicity hazards are estimated based on a decision tree approach following the above-mentioned rules. While low and intermediate oral toxicity is assigned to Class I and II substances, Class III contains substances with reactive functional groups in their structure suggesting potential significant toxicity (Cramer et al., 1978, Patlewicz et al., 2008).

## 3 Results and discussion

### 3.1 Extraction of analytes from coffee capsules

Soxhlet and SLE are two of the most used extraction techniques, partly due to their simplicity. Soxhlet extraction is considered to be very efficient even for analytes that are difficult to extract, because it can be employed for long periods. ASE allows to use typical organic solvents at temperatures higher than their boiling points due to a combination of high pressure and temperature. Therefore, it offers increased efficiency and a drastically reduced extraction time. The three techniques have been evaluated for the extraction of the oligomer content in one type of PBT-made coffee capsules. This extraction study was not exhaustive in terms of selection of extraction types, solvents/conditions of extraction, nor performed under optimized conditions; the objective was to present an initial comparison of the extracting efficiency of three commonly used methods for this specific type of materials and substances. For the same reason, dissolution of the polymers and some potential issues such as degradation of the polymer or ethanolysis of oligomers during the extraction procedures were not considered in this work.

The selection of the extracting solvent is crucial. For instance, PET dissolves completely in chlorinated solvents under the high temperature and pressure of ASE (Alberto Lopes, Tsochatzis, Emons, & Hoekstra, 2018). Moreover, chlorinated solvents are typically used to extract oligomers from PES items (Brenz et al., 2018, Tsochatzis et al., 2019b). Other options for isolating oligomers from FCM polymeric materials include the drastic complete dissolution of the PES followed by precipitation of the polymer and recuperation of the oligomers (Tsochatzis et al., 2019b). In order to select the extraction solvent for the study here, the following points were considered:

• For comparability reasons the same solvent should be used with the three extraction techniques;
• Ethanol proved to be an efficient alternative to chlorinated solvents in the ASE extraction from PES (Alberto Lopes et al., 2018);
• The use of chlorinated solvents can improve extraction yields of oligomers, but this effect is expected to be more significant when high oligomeric amounts are present in the polymer;
• The dissolution technique may lead to potential misleading results if the polymer degrades into its oligomers during the process (Tsochatzis et al., 2019c).

Considering all these aspects ethanol was selected as extraction solvent for all the three extracting protocols. The results are presented in Table 2.

**Table 2:** Mass fractions (mg kg^−1^ material) of extracted oligomers, using the three extraction methods: ASE, SLE and Soxhlet extraction with ethanol, under three detection conditions. < indicates the value of the LOD.

	ASE (mg kg^−1^)				Soxhlet (mg kg^−1^)				SLE (mg kg^−1^)			
	UV (240 nm)	FLD (λ_emm._ = 360 nm)	FLD (λ_emm._ = 320 nm)	Detector's signal RSD (%)	UV (240 nm)	FLD (λ_emm_ = 360 nm)	FLD (λ_emm._ = 320 nm)	Detector's signal RSD (%)	UV (240 nm)	FLD (λ_emm._ = 360 nm)	FLD (λ_emm._ = 320 nm)	Detector's signal RSD (%)
[TPA-EG]_2_	< *1.2 × 10^-3^*	< *0.4 × 10^-3^*	< *1.5 × 10^-3^*	–	< *1.2 × 10^-3^*	< *0.4 × 10^-3^*	< *1.5 × 10^-3^*	–	< *1.2 × 10^-3^*	< *0.4 × 10^-3^*	< *1.5 × 10^-3^*	–
[TPA-BG]_2_	1089.2	1096.4	1096.8	0.4	72.5	74.4	73.4	1.3	104.4	108.9	108.3	2.3
[TPA-EG]_3_	2.7	2.3	2.6	8.4	3.2	3.5	3.4	5.1	0.6	0.6	0.6	4.9
[TPA-EG]_4_	0.4	0.4	0.4	3.0	0.2	0.2	0.2	5.8	< *1.0 × 10^-3^*	< *1.5 × 10^-3^*	< *1.2 × 10^-3^*	*–*
[TPA-EG]_5_	< *1.0 × 10^-3^*	< *1.5 × 10^-3^*	< *1.2 × 10^-3^*	–	< *1.0 × 10^-3^*	< *1.5 × 10^-3^*	< *1.2 × 10^-3^*	–	< *1.0 × 10^-3^*	< *1.5 × 10^-3^*	< *1.2 × 10^-3^*	–
[TPA-BG]_3_	264.7	268.1	270.0	1.0	15.4	15.3	15.5	0.8	64.3	66.4	65.5	1.6
[TPA-EG]_6_	1.8	1.7	*1.5*	7.0	0.1	0.1	0.1	9.4	1.0	*0.5*	0.9	8.5
[TPA-EG]_7_	<1.6 *× 10^-3^*	*< 2.3 × 10^-3^*	*< 2.2 × 10^-3^*	–	< *1.6 × 10^-3^*	< *2.3 × 10^-3^*	< *2.2 × 10^-3^*	–	< *1.6 × 10^-3^*	< *2.3 × 10^-3^*	< *2.2 × 10^-3^*	–
[TPA-BG]_4_	0.6	0.5	0.6	9.5	0.5	0.5	0.5	3.0	1.0	1.0	1.1	6.3
[TPA-BG]_5_	3.1	3.1	3.3	2.4	0.8	0.8	0.9	9.9	2.1	2.1	2.1	1.3
Sum of cyclic oligomers	1362.5	1372.5	1375.2		92.7	94.8	94.0		173.4	179.5	178.5	

The three extraction methods showed different extraction efficiencies. ASE seems to be the most efficient method for the majority of the oligomers, except for [TPA-EG]_3_ and [TPA-BG]_4_. For [TPA-BG]_4_ SLE seems to be more efficient, whereas for [TPA-EG]_3_ Soxhlet extraction provided the highest results. However, the oligomers with higher molecular weight, namely [TPA-EG]_6_ and [TPA-EG]_7_, could almost only be extracted by using ASE. Comparing Soxhlet and SLE extractions, the latter was more efficient for a higher number of oligomers, even when the Soxhlet extraction was lasting for 48 h. A longer Soxhlet extraction procedure would not lead to a significant increase of the yields of extraction. Additionally, considering that SLE (within one day) and ASE (within 30 min) allow almost the complete extraction of the target analytes, Soxhlet is not the most suitable extraction technique.

The PBT cyclic oligomers studied in this work correspond to the FCM substance No. 885 in Regulation (EU) No. 10/2011. This substance is allowed as additive, but not as monomer, in PET, PBT, polycarbonate, polystyrene and rigid poly(vinyl chloride) plastics in mass fractions up to 1% w/w, in contact with aqueous, acidic and alcoholic foods, for long-term storage at room temperature. The total mass fraction of the PBT oligomers in the coffee capsule tested was below its legislative limit. However, the use of the coffee capsules is not in line with the related restriction. Although the materials in the coffee capsules are during long-term storage only in contact with the coffee at room temperature, the capsules could be in contact with a coffee-water mixture at temperatures of up to 100 °C during the preparation of the coffee. For the capsules studied here, the average temperature would range from 80 °C to 85 °C according to the manufacturer's manual. Therefore, whether the coffee capsules comply for FCM No 885 or not depends on the declaration of compliance (DOC) of the producer. If the DOC would state that the additive FCM No. 885 was intentionally added, then the capsules would be non-compliant. On the other hand, if the PBT oligomers were not added intentionally and the supporting documentation would contain a risk assessment of the PBT oligomers according to Article 19 of Regulation (EU) No. 10/2011, then the capsules could be compliant.

Also PET oligomers were detected in the extract of the coffee capsule. These substances are not regulated at EU level and should be considered as NIAS. Since a real coffee capsule deprived of coffee and all inner parts (including the sealing films made of PET) was used, no PET oligomers were expected to be found. Their presence in PBT may indicate a potential contamination.

A representative chromatogram of an ASE extract is presented as supplementary material (Fig. S1).

### 3.2 Optimization of the HPLC-UV/FLD method

**Fig. 1:**
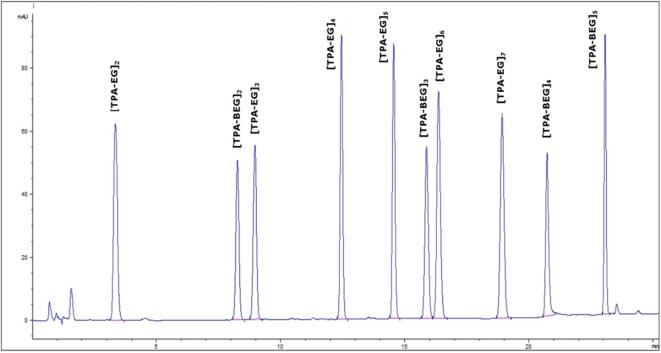
HPLC-UV (240 nm) chromatogram of food simulant C fortified with oligomer standard mixture at concentrations of 0.5 mg L^-1.^

The analytical method reported here is based on the one used in a previous work (Tsochatzis, Alberto, Dehouck, Robouch, & Hoekstra, 2019a). The chromatographic program used in that method was optimised to allow good resolution for all ten analytes, and it uses an acetonitrile/water mobile phase without any organic modifier. Initial tests were carried out with a detection at 240 nm, the best compromise for a maximum UV absorption for all oligomers. A chromatogram of the mixture is presented in Fig. 1.

The chemical structures of the PET and PBT cyclic oligomers, containing aromatic rings conjugated with carboxy groups in cyclic configuration, raised the question if a fluorescence behaviour would be present and measurable with the use of a FLD. As no data were found in the literature, some tests with varying excitation and emission wavelengths were performed using the same HPLC eluent program. Figs. S1 and S2 show examples of obtained chromatograms at different wavelengths for some of the studied oligomers.

Excitation wavelengths below 230 nm and above 270 nm were also tested but disregarded due to a significant increase of the baseline noise. For most oligomers the excitation was higher at a wavelength of 240 nm and 250 nm. However, the two dimers caused only a higher excitation signal at 250 nm, which was therefore used for the selection of the maximum emission wavelength. Fig. S2 shows that for [TPA-EG]_2_ the emission wavelength of 360 nm creates the highest excitation signal at 250 nm (also for [TPA-BG]_2_, not presented). For the other oligomers, the response varied for each oligomer, as illustrated for example in Fig. S2 for three of them. The emission at 340 nm and 360 nm produced for [TPA-EG]_5_ and [TPA-BG]_3_ a similar emission intensity and a slightly lower value at 320 nm. For [TPA-EG]_6_ an emission wavelength of 390 nm showed clearly a higher emission intensity, followed by the signal from 320 nm. As a compromise an emission wavelength of 320 nm was finally selected. The possibility of using dual channels in the fluorescence detector allowed the simultaneously monitoring with an emission wavelength of 360 nm, the most sensitive for the dimers.

The repeatability of the analytical signals from all tested detectors (UV at 240 nm, FLD with λ_emm._ of 320 and 360 nm) at different mass fraction levels had RSDs below 10% in all cases (see Tables S2 and S3), which showed that all three detection techniques are suitable.

### 3.3 Method validation

The precision of the retention times (t_R_) of the target analytes was evaluated by performing 10 consecutive injections at a concentration of 500 μg L^−1^ in spiked water and food simulant C, respectively. Linearity proved to be acceptable, as the R^2^ values were at least 0.99 for all the oligomers and detection modes used, while the residual plots did not present any trend (Table 3). The results for the UV detector were equivalent to those of the fluorescent detector. The data are also similar to those using an UHPLC-qTOF-MS method reported previously (Tsochatzis et al., 2020). All LOQs were at low μg kg^−1^ levels, ranging from 0.11 μg kg^−1^ ([TPA-BG]_2,_ FLD at λ_emm_ 360 nm) to 0.67 μg kg^−1^ ([TPA-EG]_7_, FLD at λ_emm_ 320 nm). For [TPA-EG]_2_ and [TPA-BG]_2_, the smallest of the two studied PES oligomers, the fluorescence detection at 360 nm emission showed to be more sensitive for [TPA-EG]_2_ and [TPA-BG]_2_, with LODs reaching levels of 40 pg kg^−1^. As expected, the concentration by a factor of 20 improved the detection power of the method, facilitated by the limited volatility of the substances (Tsochatzis et al., 2020).

**Table 3:** Characteristics from the analysis of PET and PBT cyclic oligomers: retention times, linearity (R^2^), linear range, LODs and LOQs.

			UV 240 nm			FLD (λ_emm_ = 360 nm)			FLD (λ_emm_ = 320 nm)		
Analyte *	Retention time ± SD** (min)	Working range (μg kg^−1^)	R^2^	LOD (μg kg^−1^)	LOQ (μg kg^−1^)	R^2^	LOD (μg kg^−1^)	LOQ (μg kg^−1^)	R^2^	LOD (μg kg^−1^)	LOQ (μg kg^−1^)
[TPA-EG]_2_	3.4 ± 0.1	0.1–2000.0	0.996	0.12	0.37	0.996	0.04	0.13	0.996	0.15	0.45
[TPA-BG]_2_	8.2 ± 0.1	0.1–2000.0	0.996	0.12	0.34	0.996	0.04	0.11	0.996	0.14	0.42
[TPA-EG]_3_	8.9 ± 0.1	0.3–2000.0	0.990	0.09	0.25	0.997	0.15	0.45	0.997	0.10	0.30
[TPA-EG]_4_	12.4 ± 0.3	0.2–2000.0	0.997	0.08	0.23	0.998	0.13	0.35	0.997	0.10	0.30
[TPA-EG]_5_	14.5 ± 0.2	0.3–2000.0	0.998	0.10	0.28	0.997	0.15	0.44	0.997	0.12	0.34
[TPA-BG]_3_	15.9 ± 0.2	0.2–2000.0	0.996	0.08	0.23	0.995	0.13	0.35	0.996	0.10	0.30
[TPA-EG]_6_	16.3 ± 0.3	0.4–2000.0	0.998	0.13	0.38	0.998	0.15	0.44	0.998	0.15	0.45
[TPA-EG]_7_	18.9 ± 0.5	0.5–2000.0	0.999	0.16	0.45	0.999	0.23	0.65	0.999	0.22	0.67
[TPA-BG]_4_	20.7 ± 0.4	0.2–2000.0	0.999	0.08	0.23	0.999	0.16	0.47	0.999	0.12	0.34
[TPA-BG]_5_	23.1 ± 0.2	0.3–2000.0	0.998	0.09	0.25	0.998	0.19	0.57	0.998	0.12	0.34

**Note:** For the transformation of concentrations (μg L^-1^) to mass fractions (μg kg^−1^) a density of 1.0 g mL^−1^ was applied.

* For chemical names of the analytes, please check abbreviation in Table S1.

** Combined average of the t_R_ for each analyte obtained with 10 replicates per spiked sample (water or food simulant C) (SD = standard deviation).

The method’s precision and accuracy were characterised by measuring the analytes at two different levels (100 and 500 ng g^−1^) using water and food simulant C tested. All the results are presented in Tables S2 and S3 of the supplementary information. Data obtained with the FLD detector were similar to the ones resulting from UV detection.

The recoveries ranged from 95% ([TPA-EG]_4_) up to 114% ([TPA-BG]_5_). Repeatability and intermediate precision expressed as RSDs was better than 12%. The range of observed recoveries can be considered as satisfactory, but indicate a partially remaining bias in the analysis. Most of the highest recovery data were observed with the heavier oligomers, which could be explained by potential solubility issues for these substances. This effect has already been found for PET oligomers in a previous work (Tsochatzis et al., 2020). The unknown uncertainty of the purity statement for the commercial PET analytical standards is another potential source for a bias (Tsochatzis et al., 2020). Nevertheless, the performance characteristics of this analytical method for the 1st series PET cyclic trimer are in line with the values reported in other publications (Begley, Dennison, & Hollifield, 1990; Kim and Lee, 2012; Tsochatzis et al., 2020).

### 3.4 Results from mimicking a consumer use of coffee capsules

At present it does not exist an official migration test protocol for coffee capsules so the migration tests were performed as described in section 2.6.

For the immersion test the worst foreseeable contact time is <30 s at a worst case temperature of 85 °C. This results in test conditions as defined in Regulation (EU) No. 10/2011, Annex V of 5 min at 100 °C or reflux. The coffee capsules were emptied, wiped carefully with a soft cotton cloth, while retaining the inner PET films and the outside aluminum-based lid.

The coffee machine test was designed in order to reflect the real conditions of use of the coffee capsules. The tested items were prepared as before, closed by pressing the aluminum lid against the capsule’s edges, and inserted into the coffee machine. From the coffee making step’ volumes of 100 mL of water and food simulant C percolated through the machine, respectively, have been collected for each capsule as to mimic the preparation of a coffee.

All the solutions obtained with both tests were analysed directly or after concentration by a factor of about 20. Results for the immersion test are presented in Table 4 and the ones for the coffee machine process in Table 4. A representative chromatogram of the mixture obtained with this test (PBT caps 1) is presented as supplementary material (Fig. S4).

**Table 4:** Migrated mass (µg) of oligomers per capsule during immersion of the coffee capsule in water and food simulant C (FS C) by immersion at boiling temperature for 5 min or in a coffee machine.

Migrated mass (µg) of oligomers per coffee capsule (immersion test)										
Oligomers	PBT caps. 1		PBT caps. 2		PP caps. 1		PP caps. 2		PP caps. 3	
	Water	FS C	Water	FS C	Water	FS C	Water	FS C	Water	FS C
**[TPA-EG]_2_**	16.2	16.5	3.2	3.2	0.7	0.7	*< 0.01*	*< 0.01*	*< 0.01*	*< 0.01*
**[TPA-EG]_3_**	0.94	28.32	1.89	53.54	0.63	22.03	12.59	125.9	0.9	14.4
**[TPA-EG]_4_**	*< 0.01*	*< 0.01*	0.02	0.51	*< 0.01*	*< 0.01*	*< 0.01*	*< 0.01*	*< 0.01*	*< 0.01*
**[TPA-EG]_6_**	1.1	1.4	0.1	0.1	*< 0.01*	*< 0.01*	0.6	0.8	0.2	0.3
**[TPA-BG]_2_**	58.1	174.2	58.1	159.7	2.9	8.7	49.4	74.1	1.45	4.36
**[TPA-BG]_3_**	0.5	13.2	0.6	13.8	1.2	25.9	1.8	44.1	0.1	2.6
**[TPA-BG]_4_**	0.03	0.18	0.02	0.15	*< 0.01*	*< 0.01*	0.04	0.83	0.03	*< 0.01*
**[TPA-BG]_5_**	0.03	0.6	0.03	0.3	0.1	4.2	0.6	6.0	1.2	17.4
**Sum of oligomers**	76.8	234.4	63.9	231.3	5.5	61.5	65.0	251.6	3.9	38.9
Migrated mass (µg) of oligomers per coffee capsule* (coffee machine)										
**Oligomers**	**PBT caps. 1**		**PBT caps. 2**		**PP caps. 1**		**PP caps. 2**		**PP caps. 3**	
	**Water**	**FS C**	**Water**	**FS C**	**Water**	**FS C**	**Water**	**FS C**	**Water**	**FS C**
**[TPA-EG]_2_**	2.5	4.7	0.5	1.1	0.1	0.2	1.1	1.8	*< 0.01*	*< 0.01*
**[TPA-EG]_3_**	0.1	1.5	0.7	14.5	0.2	0.6	3.2	12.6	0.3	1.2
**[TPA-EG]_4_**	*< 0.01*	*< 0.01*	0.04	0.02	*< 0.01*	*< 0.01*	*< 0.01*	0.1	*< 0.01*	0.1
**[TPA-EG]_6_**	2.0	0.01	0.2	*< 0.01*	*< 0.01*	*< 0.01*	1.3	0.5	0.4	0.6
**[TPA-BG]_2_**	12.2	24.7	11.5	17.8	0.4	0.8	7.1	13.5	0.2	1.1
**[TPA-BG]_3_**	0.3	0.1	0.4	0.2	1.1	0.8	1.7	1.3	0.1	0.1
**[TPA-BG]_4_**	0.1	0.1	0.04	0.1	0.02	0.04	0.5	0.7	0.2	0.3
**[TPA-BG]_5_**	0.1	0.1	0.1	0.2	1.0	1.6	0.5	0.8	1.0	1.1
**Sum of oligomers**	17.1	32.9	13.4	33.7	2.8	4.1	15.3	31.2	2.2	4.4

* Representing one coffee (100 mL).

It can be seen from Table 4 that a migration of oligomers occurred into both water and food simulant C during the immersion test. Only [TPA-EG]_5_ and [TPA-EG]_7_ were not detected in water or food simulant C with any of the capsules. The results demonstrate that migration to food simulant C was always higher than to water. The PBT capsules have caused a higher total migration of oligomers, ranging from 63.9 to 76.8 μg/capsule in water to 231–234 μg/capsule in food simulant C, compared to the total oligomer migration from PP. For two of the PP capsules, the migration to water was in the range of 3.9–5.5 μg/capsule and in food simulant C in the range of 38.0–61.5 μg/capsule. The third PP capsule has induced a similar total oligomer migration as the PBT capsules. This PP capsule originally contained soluble coffee and had only one inner PET film. It was also less rigid than the other two PP capsules.

The results of the migration during the operation of the coffee machine (Table 4) indicate that the total oligomer migration from PBT caps. 1, PBT caps. 2 and PP caps. 2 was a factor of 4–5 lower for water and a factor of 8 lower for food simulant C compared to the migration using immersion. This factor was 2–3 for both water and food simulant C in case of PP caps. 1 and PP caps. 3. This difference was expected, since the test conditions of the migration during the operation of the coffee machine are less severe, with lower temperatures (80–85 °C) and shorter exposure time (<30 s). In addition, the surface of the material in contact with water or food simulant C in the immersion test was about twice the surface exposed under real use conditions in the coffee machine. Also, no migration of [TPA-EG]_5_ and [TPA-EG]_7_ from the capsules was identified. PBT oligomers migrated with the highest amount for all studied capsules: [TPA-BG]_2_ for PBT cap 1, PBT cap 2 and PP cap 2 , and [TPA-BG]_5_ for PP cap 1 and PP cap 3. Regarding the PET cyclic oligomers, [TPA-EG]_3_ migrated with the highest amount from PBT cap 2, PP cap 1, PP cap 2 and PP cap 3, whereas [TPA-EG]_2_ migrated with the highest amount from PBT cap 1. It has to be noted that the quantification of [TPA-EG]_2_ may have been affected by interfering substances originating from coffee residues in the capsules. These substances eluted in the first minutes and may have disturbed the baseline.

Recently Eckardt et al. reported results for the *in vitro* digestibility of cyclic aromatic polyesters, namely PBT cyclic dimer and trimer and PET trimer, following a semi-quantitative approach, using terephthalic acid. They reported that cleavage of certain cyclic polyester oligomers to linear ones is occurring under human intestinal conditions, down to a plateau. The subsequent degradation of these linear products into smaller linear oligomers would again increase the probability of high intestinal absorption of the molecules (Eckardt et al., 2019).

### 3.5 Considerations related to a potential exposure

Health effects of the studied oligomers are relatively unknown, as no experimental toxicological data are available in the literature (Brenz et al., 2018, Castle and Barthélémy, 2016). An *in silico* assessment of PET oligomers indicated the absence of genotoxicity (Tsochatzis et al., 2020), but nothing has been reported for PBT cyclic oligomers. EFSA's Scientific opinion on FCM No. 885 is stating that no genotoxic properties are expected for these substances, as they “are devoid of functional groups associated with genotoxicity” (EFSA, 2019).

EFSA established a threshold of 50 μg kg^−1^ for the total oligomer (below 1000 Da) migration of new polyester co-monomer FCMs (EFSA, 2014a, EFSA, 2014b). This threshold for FCM No. 1031 and No. 1052 has been taken over as a limit for the migration in Regulation (EU) No. 10/2011 (Regulation (EU) No. 10/2011). In this study this limit is considered for a comparative evaluation of the exposure of the PET and PBT cyclic oligomers.

In the absence of toxicity data and under the assumption that there are no genotoxic effects, EFSA proposed that the TTC approach could be used to classify the risk of an individual substance (EFSA, 2012, EFSA, 2016). Applying this approach, it appears that all the oligomers studied in this work may be classified as Cramer class III substances and consequently a maximum exposure level of 90 μg/person/day could be assigned.

**Fig. 2:**
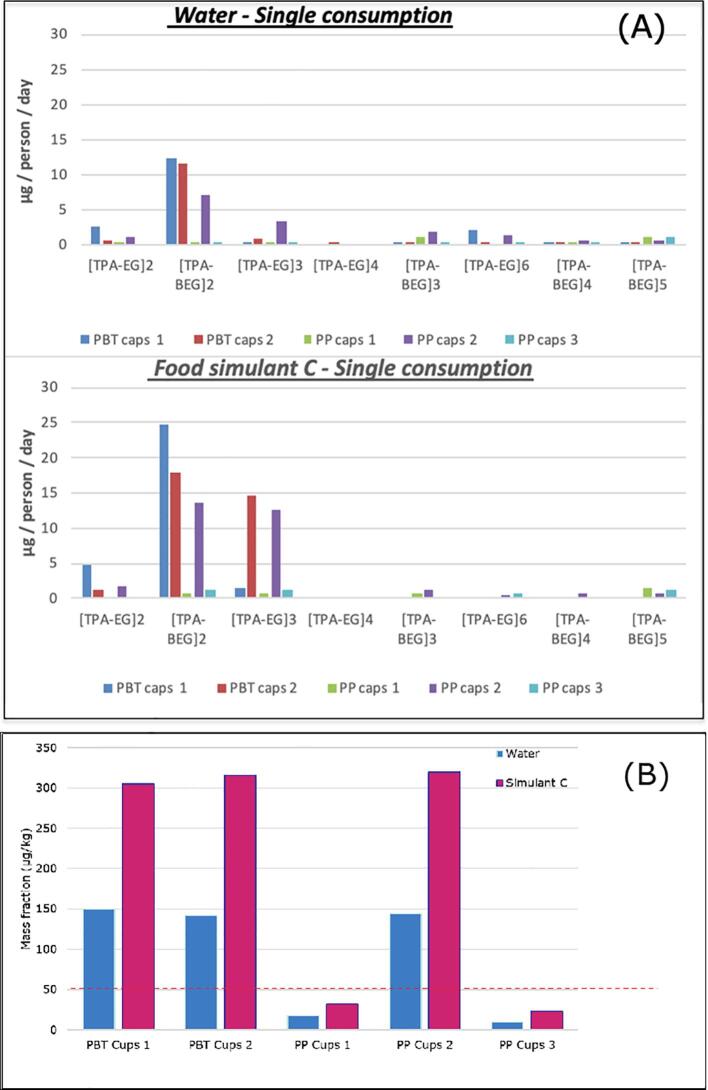
Exposure estimate (A) individual PET and PBT oligomers (/person/day) and (B) the sum of quantified mass fractions (µg kg^−1^) of PET and PBT oligomers (M < 1000 Da), in water and food simulant C.

The results from migration experiments in water and food simulant C obtained with the coffee machine are presented in Fig. 2 in relation to both exposure approaches. For more coffees per day the use of the same type of capsules is assumed. For the purpose of this study only the oligomers migrating from the coffee capsules were considered. As the ground coffee was removed from the capsules prior to the experiments, oligomers that could have already migrated from the capsule to the coffee during storage were discarded. Therefore, real exposure may be higher than calculated below.

It can be seen from Fig. 2A that the consumption of a single coffee per day does not exceeds the TTC limit of 90 µg/person/day, for both water and food simulant C. When multiple consumption of 5 coffees per day are considered, the amounts of all the PET and PBT cyclic oligomers remain below the threshold, with the exception of [TPA-BG]_2_ migrating from sample PBT-1.

Fig. 2B shows the total mass fraction of oligomers with a MW of <1000 Da ([TPA-EG]_6_ and [TPA-BG]_5_ excluded) present in water and food simulant C. It can be seen that the single consumption in water is already much higher than the 50 µg/kg food for three out of the five studied capsules. The results presented in this work underline the need to establish toxicological data for this type of oligomers and to have a harmonised risk assessment approach.

## 4 Conclusions

The developed HPLC method allows in a simple and reliable manner the simultaneous analysis of 10 cyclic oligomers from PET and PBT, two of the most widely used PES in FCMs. It was applied for the analysis of migration solutions originating from a few market relevant coffee capsules made of PBT and PP and to organic solvent extracts of the capsules. To the best of the authors’ knowledge, it is the first time that such items have been analysed for the presence of oligomers.

The sum of the mass fractions of the PBT oligomers was tested in one coffee capsule and complied with the restriction of the mass fraction limit of FCM substance No. 885 in the polymer laid down in Regulation (EU) No. 10/2011. However, the foreseeable use conditions of the coffee capsules are not compliant with the restriction of FCM substance No. 885. The coffee capsule might be only compliant, if the PBT and PET oligomers were present unintentionally and if the risk of their migration is assessed and documented in the declaration of compliance of the producer according to Article 19 of Regulation (EU) No. 10/2011.

The risk from PBT oligomers was estimated by applying two approaches. The TTC approach indicates that the risk of drinking one cup of coffee from the tested capsules is well below the threshold for Cramer class III substances. Following another approach with assuming a total specific migration limit of 50 µg kg^−1^ food for oligomers with a MW lower than 1000 Da as it was proposed by EFSA for other polyester co-polymers and set in Regulation (EU) No. 10/2011, three out of the five coffee capsules surpassed this limit. In conclusion, the risk from PBT oligomers consumption remains open and further toxicological studies are needed.

Disclaimer:

Certain commercial equipment, instruments, and materials are identified in this paper/report to specify adequately the experimental procedure. In no case does such identification imply recommendation or endorsement by the European Commission, nor does it imply that the material or equipment is necessarily the best available for the purpose.

## CRediT authorship contribution statement

**Joao Alberto Lopes:** Conceptualization, Methodology, Validation, Formal analysis, Investigation, Data curation, Writing - original draft, Writing - review & editing. **Emmanouil D. Tsochatzis:** Conceptualization, Methodology, Validation, Formal analysis, Investigation, Writing - review & editing, Data curation, Writing - original draft. **Lubomir Karasek:** Investigation, Writing - original draft, Writing - review & editing. **Eddo J. Hoekstra:** Writing - review & editing, Project administration. **Hendrik Emons:** Writing - review & editing, Project administration.

## Declaration of Competing Interest

The authors declare that they have no known competing financial interests or personal relationships that could have appeared to influence the work reported in this paper.
