# Supplementary material for: Analysis of PBT and PET cyclic oligomers in extracts of coffee capsules and food simulants by a HPLC-UV/FLD method
Source: Food Chem. 2021 May 30;345:128739. doi: 10.1016/j.foodchem.2020.128739 (PMC7896039; doi:10.1016/j.foodchem.2020.128739)
Supplement: Supplementary data 1 [file mmc1.docx]

**Analysis of polybutylene and polyethylene cyclic oligomers in coffee capsules extracts and food simulants by HPLC-DAD/FLD method**

Joao Alberto Lopes^1^, Emmanouil D. Tsochatzis^1^*, Lubomir Karasek^1^, Eddo J. Hoekstra^2^ and Hendrik Emons^1^

^1^European Commission, Joint Research Centre (JRC), Geel, Belgium.

^1^European Commission, Joint Research Centre (JRC), Ispra, Italy.

**Supplementary Info**

**
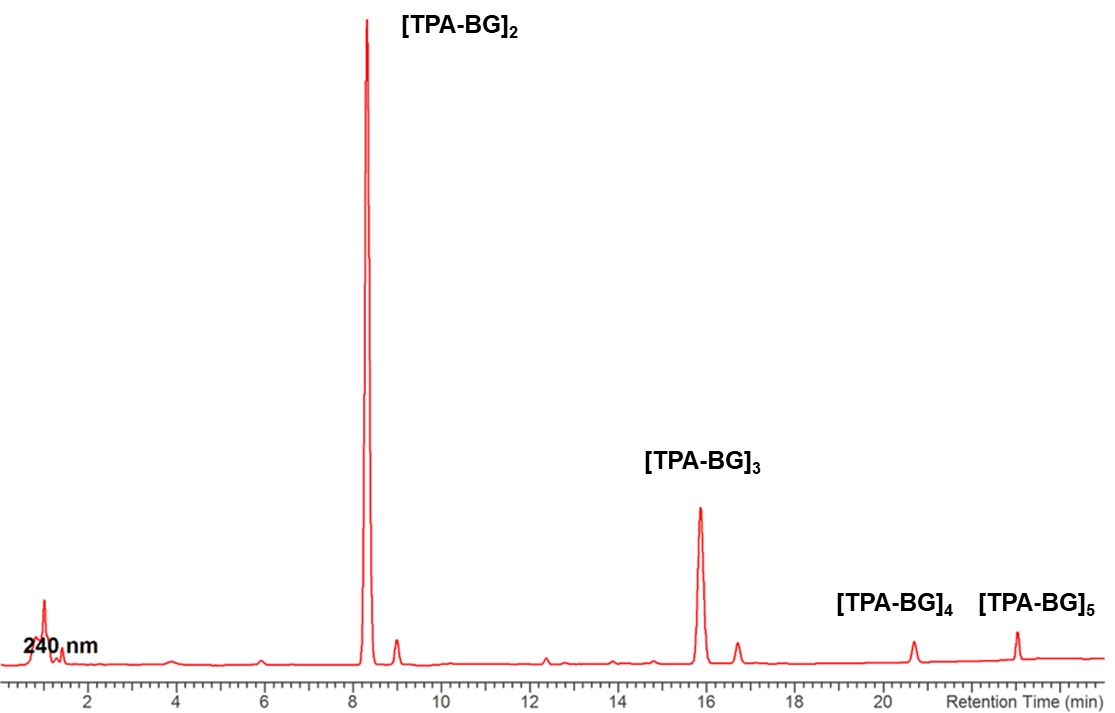
**

**Figure S1.** HPLC-UV (240 nm) chromatogram of PBT ASE extract.


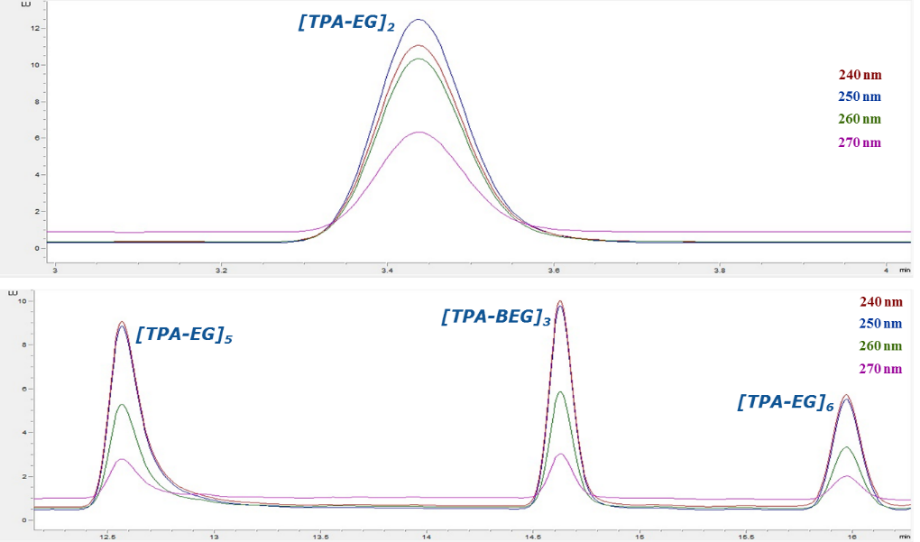


**Figure S2.** Overlay of the FLD signal of a 5 mg L^-1^ oligomers’ standard mixture, excitation ranging from 240 to 270 nm, emission kept at 320 nm


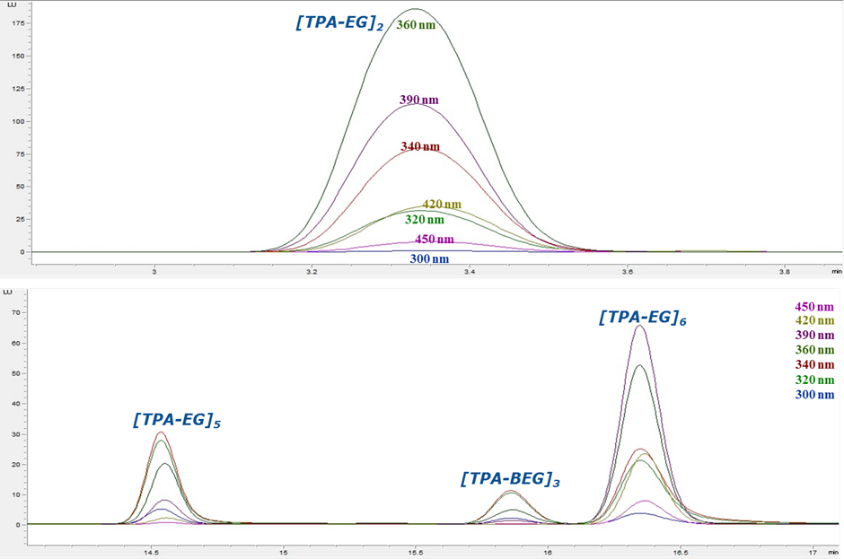


**Figure S3.** Overlay of the FLD signal of a 5 mg L^-1^ oligomers’ standard mixture, excitation was kept at 250 nm, emission ranging from 300 to 450 nm

**
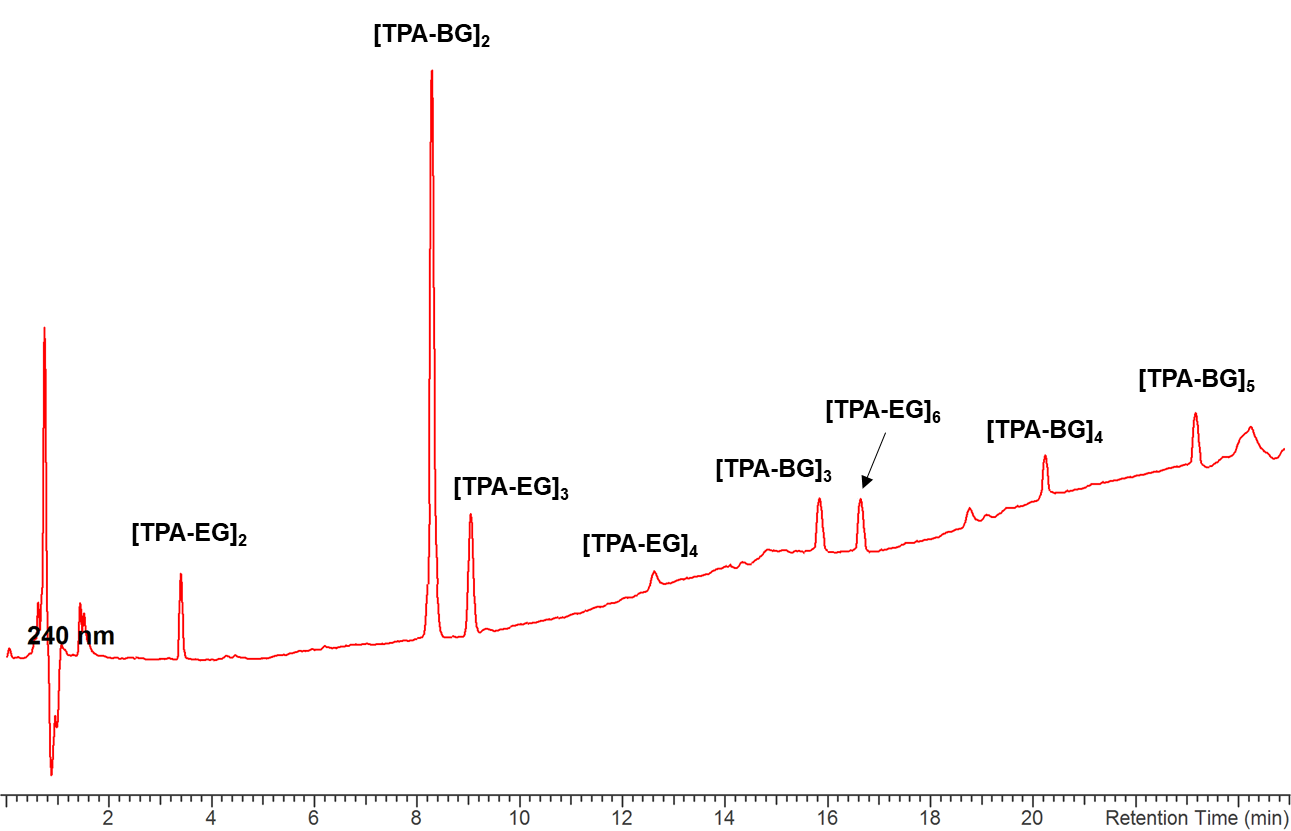
**

**Figure S4.** HPLC-UV (240 nm) chromatogram of food simulant C after the immersion test with capsule PBT1

**Table S1 –** Chemical information of the studied oligomers

| **Chemical**  **name** | **Acronym** | **Chemical**  **Formula** | **CAS No.** | **MW (g/mol)** |
| --- | --- | --- | --- | --- |
| Ethylene Terephthalate Cyclic Dimer * | [TPA-EG]_2_ | C₂₀H₁₆O₈ | 24388-68-9 | 384.3 |
| Butylene Terephthalate Cyclic Dimer** | [TPA-BG]_2_ | C₂₄H₂₄O₈ | 63440-93-7 | 440.4 |
| Ethylene Terephthalate Cyclic Trimer * | [TPA-EG]_3_ | C₃₀H₂₄O₁₂ | 7441-32-9 | 576.5 |
| Ethylene Terephthalate Cyclic Tetramer* | [TPA-EG]_4_ | C₄₀H₃₂O₁₆ | 16104-96-4 | 768.7 |
| Ethylene Terephthalate Cyclic Pentamer * | [TPA-EG]_5_ | C₅₀H₄₀O₂₀ | 16104-97-5 | 960.8 |
| Butylene Terephthalate Cyclic Trimer ** | [TPA-BG]_3_ | C₃₆H₃₆O₁₂ | 63440-94-8 | 660.7 |
| Ethylene Terephthalate Cyclic Hexamer * | [TPA-EG]_6_ | C₆₀H₄₈O₂₄ | 29644-29-9 | 1153.0 |
| Ethylene Terephthalate Cyclic Heptamer * | [TPA-EG]_7_ | C₇₀H₅₆O₂₈ | 29668-12-0 | 1345.2 |
| Butylene Terephthalate Cyclic Tetramer ** | [TPA-BG]_4_ | C₄₈H₄₈O₁₆ | 29278-72-6 | 880.9 |
| Butylene Terephthalate Cyclic Pentamer ** | [TPA-BG]_5_ | C₆₀H₆₀O₂₀ | 82298-33-7 | 1101.1 |

* Oligomers considered as Non-Intentional Added Substances (NIAS) in FCMs

** Part of mixture FCM No. 885 from the positive list of Regulation (EU) No. 10/2011

**Table S2.** Precision and trueness results for the analysis of oligomers in fortified water samples

| **Oligomer** | **Repeatability (n=6)** | | | | | | | |
| --- | --- | --- | --- | --- | --- | --- | --- | --- |
|  | **1^st^ Mass fraction level** | | | | **2^nd^ Mass fraction level** | | | |
|  | **Added** | **Found**  (μg kg^-1^) | **RSD** | **Recovery** | **Added** | **Found**  (μg kg^-1^) | **RSD** | **Recovery** |
|  | (μg kg^-1^) | **Average (±Sd)** | **(%)** | **(%)** | (μg kg^-1^) | **Average (±Sd)** | **(%)** | **(%)** |
| [TPA-EG]_2_ | 100 | 103.3 ± 1.7 | 1.6 | 103.3 | 500 | 503.1 ± 7.4 | 1.5 | 100.6 |
| [TPA-BG]_2_ |  | 102.5 ± 3.9 | 3.8 | 102.5 |  | 503.7 ± 10.4 | 2.1 | 100.7 |
| [TPA-EG]_3_ |  | 105.2 ± 3.1 | 2.9 | 105.2 |  | 509.4 ± 3.0 | 1.7 | 101.9 |
| [TPA-EG]_4_ |  | 96.2 ± 3.0 | 3.1 | 96.2 |  | 495.6 ± 6.7 | 1.4 | 99.1 |
| [TPA-EG]_5_ |  | 104.3 ± 2.8 | 2.7 | 104.3 |  | 502.6 ± 4.5 | 0.9 | 100.5 |
| [TPA-BG]_3_ |  | 103.4 ± 2.3 | 2.2 | 103.4 |  | 500.4 ± 5.2 | 1.0 | 100.1 |
| [TPA-EG]_6_ |  | 107.2 ± 4.3 | 4.0 | 107.2 |  | 522.8 ± 6.6 | 1.3 | 104.6 |
| [TPA-EG]_7_ |  | 114.8 ± 6.3 | 4.3 | 106.9 |  | 529.3 ± 13.1 | 2.5 | 105.9 |
| [TPA-BG]_4_ |  | 107.5 ± 3.5 | 3.3 | 107.5 |  | 518.5 ± 17.8 | 3.4 | 103.7 |
| [TPA-BG]_5_ |  | 113.7 ± 8.6 | 4.3 | 106.3 |  | 530.4 ± 10.7 | 2.0 | 106.1 |
|  | **Intermediate precision (n=3)** | | | | | | | |
| **Oligomer** | **1^st^ Mass fraction level** | | | | **2^nd^ Mass fraction level** | | | |
|  | **Added** | **Found**  (μg kg^-1^) | **RSD** | **Recovery** | **Added** | **Found**  (μg kg^-1^) | **RSD** | **Recovery** |
|  | (μg kg^-1^) | **Average (±Sd)** | **(%)** | **(%)** | (μg kg^-1^) | **Average (±Sd)** | **(%)** | **(%)** |
| [TPA-EG]_2_ | 100 | 109.1 ± 5.6 | 5.1 | 109.1 | 500 | 531.1 ± 16.5 | 3.1 | 106.2 |
| [TPA-BG]_2_ |  | 112.4 ± 11.2 | 6.7 | 112.4 |  | 522.3 ± 19.5 | 3.7 | 104.5 |
| [TPA-EG]_3_ |  | 113.3 ± 8.1 | 7.2 | 113.3 |  | 528.1 ± 17.1 | 3.2 | 105.6 |
| [TPA-EG]_4_ |  | 103.2 ± 4.4 | 4.3 | 103.2 |  | 526.1 ± 20.8 | 3.9 | 105.2 |
| [TPA-EG]_5_ |  | 108.7 ± 7.4 | 6.8 | 108.7 |  | 523.3 ± 15.4 | 2.9 | 104.7 |
| [TPA-BG]_3_ |  | 108.4 ± 5.7 | 5.2 | 108.4 |  | 524.1 ± 16.4 | 3.1 | 104.8 |
| [TPA-EG]_6_ |  | 112.9 ± 6.7 | 6.0 | 112.9 |  | 535.9 ± 21.5 | 4.0 | 107.2 |
| [TPA-EG]_7_ |  | 111.3 ± 10.5 | 9.4 | 111.3 |  | 540.9 ± 26.6 | 4.9 | 108.2 |
| [TPA-BG]_4_ |  | 113.1 ± 8.3 | 7.3 | 113.1 |  | 539.8 ± 26.6 | 4.9 | 108.0 |
| [TPA-BG]_5_ |  | 113.7 ± 9.3 | 8.1 | 113.7 |  | 551.7 ± 37.8 | 6.9 | 110.3 |

**Note:** For the transformation of concentrations (μg L^-1^) to mass fractions (μg kg^-1^), a density of 1.0 g mL^-1^, was applied.

**Table S3.** Precision and trueness results for the analysis of oligomers in fortified food simulant C samples

| **Oligomer** | **Repeatability (n=6)** | | | | | | | | | | | | | | | |
| --- | --- | --- | --- | --- | --- | --- | --- | --- | --- | --- | --- | --- | --- | --- | --- | --- |
|  | **1^st^ Concentration level** | | | | | | | | **2^nd^ Concentration level** | | | | | | | |
|  | **Added** | | **Found**  (μg kg^-1^) | | **RSD** | | **Recovery** | | **Added** | | **Found**  (μg kg^-1^) | | **RSD** | | **Recovery** | |
|  | (μg kg^-1^) | | **Average (±Sd)** | | **(%)** | | **(%)** | | (μg kg^-1^) | | **Average (±Sd)** | | **(%)** | | **(%)** | |
| [TPA-EG]_2_ | 100 | | 102.9 ± 1.9 | | 1.9 | | 102.9 | | 500 | | 505.9 ± 8.4 | | 1.7 | | 101.2 | |
| [TPA-BG]_2_ |  |  | 103.6 ± 3.7 | | 3.5 | | 103.6 | |  |  | 508.0 ± 9.2 | | 1.8 | | 101.6 | |
| [TPA-EG]_3_ |  |  | 106.3 ± 2.9 | | 2.8 | | 106.3 | |  |  | 512.7 ± 13.7 | | 2.7 | | 102.5 | |
| [TPA-EG]_4_ |  |  | 102.8 ± 4 | | 3.9 | | 102.1 | |  |  | 508.7 ± 11.5 | | 2.3 | | 101.7 | |
| [TPA-EG]_5_ |  |  | 106.4 ± 2.1 | | 2.0 | | 106.4 | |  |  | 496.9 ± 12.1 | | 2.4 | | 99.4 | |
| [TPA-BG]_3_ |  |  | 104.9 ± 4.4 | | 4.2 | | 104.9 | |  |  | 495.7 ± 11.3 | | 2.3 | | 99.1 | |
| [TPA-EG]_6_ |  |  | 107.5 ± 10.7 | | 10.0 | | 107.5 | |  |  | 521.5 ± 14.2 | | 2.7 | | 104.3 | |
| [TPA-EG]_7_ |  |  | 110.8 ± 10.6 | | 4.6 | | 109.8 | |  |  | 521.7 ± 16.4 | | 3.1 | | 104.3 | |
| [TPA-BG]_4_ |  |  | 110.2 ± 9.7 | | 8.8 | | 110.2 | |  |  | 529.4 ± 22.6 | | 4.3 | | 105.9 | |
| [TPA-BG]_5_ |  |  | 95.3 ± 5.3 | | 5.5 | | 95.3 | |  |  | 542.9 ± 23.3 | | 4.3 | | 108.6 | |
|  | **Intermediate precision (n=3)** | | | | | | | | | | | | | | | |
| **Oligomer** | **1^st^ Mass fraction level** | | | | | | | | **2^nd^ Mass fraction level** | | | | | | | |
|  | **Added** | **Found**  (μg kg^-1^) | | **RSD** | | **Recovery** | | **Added** | | **Found**  (μg kg^-1^) | | **RSD** | | **Recovery** | |  |
|  | (μg kg^-1^) | **Average (±Sd)** | | **(%)** | | **(%)** | | (μg kg^-1^) | | **Average (±Sd)** | | **(%)** | | **(%)** | |  |
| [TPA-EG]_2_ | 100 | 105.1 ± 5.3 | | 5.0 | | 105.1 | | 500 | | 516.8 ± 19.8 | | 3.8 | | 103.4 | |  |
| [TPA-BG]_2_ |  | 111.4 ± 9.9 | | 9.9 | | 111.4 | |  |  | 534.6 ± 3 | | 6.2 | | 106.9 | |  |
| [TPA-EG]_3_ |  | 112.6± 9.2 | | 8.1 | | 112.6 | |  |  | 546.4 ± 32.1 | | 5.9 | | 109.3 | |  |
| [TPA-EG]_4_ |  | 106.9 ± 10.4 | | 9.7 | | 106.9 | |  |  | 532.1 ± 32.9 | | 6.2 | | 106.4 | |  |
| [TPA-EG]_5_ |  | 112.4 ± 13.1 | | 11.7 | | 112.4 | |  |  | 548.3 ± 37.8 | | 6.9 | | 109.7 | |  |
| [TPA-BG]_3_ |  | 108.1 ± 8 | | 7.4 | | 108.1 | |  |  | 517.5 ± 24.8 | | 4.8 | | 103.5 | |  |
| [TPA-EG]_6_ |  | 109.8 ± 10.3 | | 9.4 | | 109.8 | |  |  | 540.6 ± 19.9 | | 3.7 | | 108.1 | |  |
| [TPA-EG]_7_ |  | 109.4 ± 8.5 | | 7.8 | | 109.4 | |  |  | 542.8 ± 8.8 | | 8.8 | | 108.6 | |  |
| [TPA-BG]_4_ |  | 111.7 ± 9.8 | | 9.8 | | 111.7 | |  |  | 564.1 ± 30 | | 5.3 | | 112.8 | |  |
| [TPA-BG]_5_ |  | 104.1 ± 10.8 | | 10.4 | | 104.1 | |  |  | 558.4 ± 39.1 | | 7.0 | | 111.7 | |  |

**Note:** For the transformation of concentrations (μg L^-1^) to mass fractions (μg kg^-1^), a density of 1.0 g mL^-1^, was applied.
